# Exposure to Yeast Shapes the Intestinal Bacterial Community Assembly in Zebrafish Larvae

**DOI:** 10.3389/fmicb.2018.01868

**Published:** 2018-08-14

**Authors:** Prabhugouda Siriyappagouder, Jorge Galindo-Villegas, Jep Lokesh, Victoriano Mulero, Jorge M. O. Fernandes, Viswanath Kiron

**Affiliations:** ^1^Faculty of Biosciences and Aquaculture, Nord University, Bodø, Norway; ^2^Department of Cell Biology and Histology, Faculty of Biology, Institute of Biomedical Research of Murcia-Arrixaca, University of Murcia, Murcia, Spain

**Keywords:** yeast, microbiota, zebrafish, germ-free, 16S rRNA, amplicon sequencing, *Debaryomyces*, *Pseudozyma*

## Abstract

Establishment of the early-life gut microbiota has a large influence on host development and succession of microbial composition in later life stages. The effect of commensal yeasts - which are known to create a conducive environment for beneficial bacteria - on the structure and diversity of fish gut microbiota still remains unexplored. The present study examined the intestinal bacterial community of zebrafish (*Danio rerio*) larvae exposed to two fish-derived yeasts by sequencing the V4 hypervariable region of bacterial 16S rRNA. The first stage of the experiment (until 7 days post-fertilization) was performed in cell culture flasks under sterile and conventional conditions for germ-free (GF) and conventionally raised (CR) larvae, respectively. The second phase was carried out under standard rearing conditions, for both groups. Exposure of GF and CR zebrafish larvae to one of the yeast species *Debaryomyces* or *Pseudozyma* affected the bacterial composition. Exposure to *Debaryomyces* resulted in a significantly higher abundance of core bacteria. The difference was mainly due to shifts in relative abundance of taxa belonging to the phylum Proteobacteria. In *Debaryomyces*-exposed CR larvae, the significantly enriched taxa included beneficial bacteria such as *Pediococcus* and *Lactococcus* (Firmicutes). Furthermore, most diversity indices of bacterial communities in yeast-exposed CR zebrafish were significantly altered compared to the control group. Such alterations were not evident in GF zebrafish. The water bacterial community was distinct from the intestinal microbiota of zebrafish larvae. Our findings indicate that early exposure to commensal yeast could cause differential bacterial assemblage, including the establishment of potentially beneficial bacteria.

## Introduction

Early microbial colonization of the gastrointestinal tract plays a key role in immunological and metabolic development of the host, which influences their health and disease status later in life (Rodríguez et al., 2015; Tanaka and Nakayama, 2017). Host–microbe coevolution has resulted in parasitic, commensal, or mutualistic relationships between the gut microbial community members and their host (Ley et al., 2008). These complex relationships are shaped by the indigenous intestinal microbial communities, which include bacteria, fungi, viruses, and protozoans (Barko et al., 2018). The initial microbial colonizers are essential for the development of neonatal gut epithelial barrier functions, maturation, and modulation of innate and adaptive immune responses (Hooper and Gordon, 2001). In addition, they may provide favorable conditions for subsequent colonization of microbial members (Houghteling and Walker, 2015) and create the platform for establishment of persistent microbial communities (Faith et al., 2013; Houghteling and Walker, 2015). Thus, the microbial composition in adults may reflect the history of early-life events, such as initial encounter with microbes, colonization and establishment of microbes in the gastrointestinal tract (Rodríguez et al., 2015).

Microbial colonization of the fish gut during larval stages is known to occur during either active uptake of water or the time of first feeding (Reitan et al., 1998; Vadstein et al., 2013). Microbes associated with chorion of fish eggs, surrounding water, and early diet influence the composition of the larval gut microbiota (Llewellyn et al., 2014). This community is further shaped by host ontogeny stage, habitat (Stephens et al., 2016) and the gut environmental conditions such as availability of nutrients, pH, and digestive enzymes (Hansen and Olafsen, 1999). The diversity of fish gut microbiota is affected by early-life factors including life stages, diet, and rearing environment on fish gut microbiota composition (Romero et al., 2014; Lokesh et al., 2018). In this context, probiotics are microorganisms that are administered in diets to promote host health and potentially modulate the gut microbiota throughout fish ontogeny from larvae to adult (Montalban-Arques et al., 2015; Ringø et al., 2016). Improving the beneficial microbial community, by altering gut microbial composition through dietary administration of probiotics, will increase feed efficiency and growth, and to maintain the health and wellbeing of aquatic animals (Llewellyn et al., 2014). For instance, lactic acid bacteria (LAB) are considered as probiotic candidates in aquaculture (Llewellyn et al., 2014) because they provide nutritional benefits and protect the host from pathogenic infections (Balcázar et al., 2007; Martínez Cruz et al., 2012; Hai, 2015).

The effect of application of yeast and their derivatives as probiotics, immunostimulants, and feed supplements is also well-documented in different animals including fish (Navarrete and Tovar-Ramírez, 2014; Vohra et al., 2016). For example, European sea bass (*Dicentrarchus labrax*) larvae fed live yeast *Debaryomyces hansenii* had improved growth, feed efficiency, survival, and immune competence (Tovar-Ramírez et al., 2004, 2010). Additionally, feeding juvenile European sturgeon (*Huso huso*) with a live yeast-supplemented diet increased the proportion of LAB in the gut (Hoseinifar et al., 2011), even if their significance for the host remains to be elucidated (Hoseinifar et al., 2011). Nevertheless, the presence of different LAB strains could have a positive effect on host; they exert different competition mechanisms (hydrogen peroxide, lactic acid, bacteriocin-like molecules) to inhibit growth of pathogens and stimulate the host immune system (Ringø and Gatesoupe, 1998; Verschuere et al., 2000; Campana et al., 2017). In a recent study, it was reported that feeding juvenile rainbow trout (*Oncorhynchus mykiss*) with functional diet incorporated with a probiotic yeast (*Saccharomyces cerevisiae*) changed the intestinal microbiota (Gonçalves and Gallardo-Escárate, 2017). Rather than allochthonous microorganisms, autochthonous microorganisms are potential functional food ingredients for modulating the composition of microbiota (O’Toole and Cooney, 2008).

To date, there is limited information about the impact of yeast on the establishment of the gut bacterial community in fish, particularly during early developmental stages. Nevertheless, it is known that the gut microbiota in fish larvae is less stable than in adults (Gatesoupe, 1999). Hence, yeast exposure during early ontogeny has the potential to modify the composition and improve the functionality of gut microbiota in fish larvae. We hypothesized that early exposure to yeast may alter the fish gut microbiota. To test this hypothesis, we explored the potential of two yeast species *Debaryomyces* sp. and *Pseudozyma* sp., which were originally isolated from Atlantic salmon (*Salmo salar*) and zebrafish (*Danio rerio*), respectively. *Debaryomyces* is frequently associated with fish and it has been considered as an excellent probiotic candidate because of its beneficial and therapeutic properties (Navarrete and Tovar-Ramírez, 2014). On the other hand, though *Pseudozyma* sp. is not commonly reported in fish, *P. fusiformata* has been detected in the gut of wild salmonids (Raggi et al., 2014). We investigated the effect of early yeast exposure on the intestinal microbiota composition of zebrafish larvae raised in germ-free (GF) or conventional conditions by sequencing the V4 hypervariable region of the bacterial 16S rRNA gene. Our findings provide the first evidence that fish-derived yeast influence the assembly of the bacterial communities during early life that could lead to a healthy gut environment in fish.

## Materials and Methods

### Yeast Strains and Culture Conditions

*Debaryomyces* sp. and *Pseudozyma* sp. used in this study were originally isolated from the intestine of Atlantic salmon and zebrafish, respectively, at Nord University, Bodø. The isolated yeast colonies were identified by PCR amplification and Sanger sequencing of the internal transcribed spacer 2 (ITS2) region of fungal rDNA. Pure cultures of the yeasts were prepared and stored in 30% glycerol (Sigma-Aldrich St. Louis, MO, United States) at -80°C. Prior to use, the cultures were revived on yeast extract peptone dextrose agar (Sigma-Aldrich) plate and broth supplemented with 0.025% chloramphenicol (Sigma-Aldrich). They were further grown in yeast extract peptone dextrose broth at 28°C, shaking the broth at 180–200 rpm for 24 h. The cultured yeast cells were harvested by centrifugation at 10,000 rpm for 10 min. Subsequently, the harvested cells were washed and resuspended in sterile phosphate-buffered saline (PBS) to obtain a final concentration of 2 × 10^5^ CFU/ml for the following exposure study.

### Ethics Statement

The experiments performed comply with the Guidelines of the European Union Council (Directive 2010/63/EU) and the Spanish RD 53/2013. Experiments and procedures were performed as approved by the Bioethical Committee of the University of Murcia (approval numbers #537/2011, #75/2014, and #216/2014).

### Zebrafish Husbandry and Preparation of Larvae

The experiments were conducted at the laboratories of the Department of Cell Biology and Histology, University of Murcia. Standard husbandry procedures (Westerfield, 2007) were followed to maintain wild type zebrafish in a re-circulation Fish-box system (Aqua Medic GmBH, Bissendorf, Germany). Fish were offered a commercial diet (GEMMA Micro 300, Skretting, Burgos, Spain) and *Artemia salina* nauplii, two times a day. Adult zebrafish (1 male: 2 female) were introduced into 1 L breeding tanks with dividers that kept the males and females apart overnight. The following morning the dividers were removed to allow natural spawning of fish. The eggs were evenly split into two groups; one-half of the eggs were used to generate GF embryos as described by Galindo-Villegas et al. (2012). Subsequently, GF embryos were reared in sterile, vented tissue culture flasks containing autoclaved and filtered egg water without antibiotics. The remaining half of the collected eggs were conventionally raised (CR) following same strategy but using regular embryo medium as described elsewhere (Galindo-Villegas et al., 2012). Both groups were carefully monitored daily, and dead eggs, if any, were aseptically removed. In addition, 50% media was daily replaced in each flask according to the respective initial conditions. The sterility of GF fish water media was daily confirmed by visual assessment of inoculated agar plates for microbial growth, as well as by conventional PCR using V4f and V4r primer pairs targeting the V4 region of the bacterial 16S rRNA (Kozich et al., 2013).

### Design of the Yeast Exposure Study

The experiments employing GF and CR zebrafish larvae were done in two phases. The first stage (from 0 to 7 days post-fertilization, dpf) was performed in experimental flasks under sterile or conventional conditions for GF and CR larvae, respectively. The second phase was carried out from 7 to 14 dpf using 1.5 L tanks under standard rearing conditions, for both groups. These tanks received water from the zebrafish recirculation system. The larvae did not receive any feed during the first phase. They were offered a commercial larval starter microfeed (NovoTom Artemia, JBL, Neuhofen, Germany) from 7 until 14 dpf, i.e., the second phase. The rearing conditions in the flasks/tanks were: temperature 28.0 ± 0.5°C, pH 7.5 and 12 h light/ 12 h dark cycle.

**FIGURE 1:**
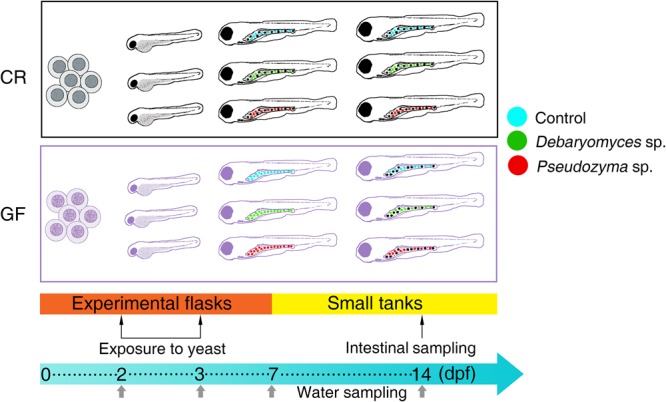
Design of the yeast exposure study. The alterations in the intestinal bacterial communities of conventionally raised (CR) and germ-free (GF) zebrafish larvae were examined after exposing them to the yeasts *Debaryomyces* sp. or *Pseudozyma* sp. for 24 h. The larvae in the control group were exposed to PBS. The intestine samples were collected on day 14 and water samples from their respective flasks/tanks were collected at days 2, 3, 7, and 14.

Zebrafish larvae, both GF and CR, were aseptically divided into three treatment groups (Control, *Debaryomyces*, and *Pseudozyma*) (**Figure 1**). Each group of larvae was maintained in triplicate flasks, prior to immersion-exposure to yeasts. At 2 dpf, GF and CR zebrafish larvae were exposed to 2 × 10^5^ CFU/ml *Debaryomyces* sp. or *Pseudozyma* sp. and incubated for 24 h (until 3 dpf) at 28°C. On the other hand, the control larvae were exposed to PBS. After the 24 h exposure, larvae were washed (3×) with regular embryo medium and raised in system water (CR) or gnotobiotic medium (GF) until 7 dpf. Rearing media were aseptically replaced daily with fresh media. At 7 dpf, larvae were transferred to small tanks, where they were raised until 14 dpf.

The different study groups and their abbreviations are: CRC, conventionally raised control; CRD, conventionally raised *Debaryomyces-*exposed; CRP, conventionally raised *Pseudozyma*-exposed; GFC, germ-free control; GFD, germ-free *Debaryomyces*-exposed; GFP, germ-free *Pseudozyma*-exposed. For water samples of the different groups, we followed the same nomenclature as above, but inserted the letter “W.” One extra group representing zebrafish system water, ZSW, was also included for comparison.

### Collection of Intestine and Water Samples

At 14 dpf, zebrafish larvae were euthanized with an overdose of MS222 (Sigma-Aldrich, Madrid, Spain) and rinsed with sterile water before dissection. The gastrointestinal tract of each laterally placed larva was aseptically excised under a Leica MZ16F fluorescence stereo microscope (Leica Microsystems, Wetzlar, Germany). The intestinal content was gently scraped off with Dumont #5 fine forceps (Fine Science Tools GmbH, Heidelberg, Germany). Each sample was a pool of intestines from 7 larvae; 2 to 3 samples were collected from each tank, making a total of 6 to 9 samples per treatment group. All samples were immediately frozen using dry ice and stored at -80°C until DNA extraction. Water samples from 3 replicate flasks or tanks for each treatment group were pooled and filtered using 0.2 μm pore size filter (Merck, Darmstadt, Germany) and stored at -80°C until use. In addition, we obtained water samples from the zebrafish facilities, which were also filtered and stored at -80°C before DNA extraction.

### Isolation of DNA, PCR Amplification and Sequencing

Genomic DNA from the pooled intestinal samples was extracted using the QIAamp Fast DNA Stool Mini Kit (Qiagen, Hilden, Germany) according to the manufacturer’s instructions, with minor modifications as described in our earlier publication (Siriyappagouder et al., 2018). In addition, we used the QIAamp DNA micro spin column instead of QIAamp column to purify and elute the DNA. DNA from water samples was extracted with the Metagenomic DNA isolation kit for water (Epicentre, Madison, WI, United States). The extracted DNA was stored at -20°C until further analyses.

To profile bacterial communities, the V4 hypervariable region (∼250 base pairs) of bacterial 16S rRNA gene was amplified using the primer pair V4f and V4r attached with Illumina barcoded adapters and sample-specific indices (Kozich et al., 2013). PCR reactions were performed in a reaction volume of 20 μl containing 13 μl of KAPA KAPA HiFi HotStart ReadyMix (2X) (KAPA Biosystems, Woburn, MA, United States), 0.3 μM of each primer, 3 μl DNA template and sterile water up to 20 μl. Distilled water and genomic DNA from the bacterial mock community (ZymoBIOMICS^TM^ Microbial Community Standard, Zymo research, Irvine, CA, United States) were used as negative and positive controls, respectively. The DNA from each sample was independently amplified in duplicate reactions in a thermocycler (Bio-Rad Laboratories, Inc., Hercules, CA, United States) with the following thermocycling conditions: initial denaturation at 95°C for 5 min, followed by 35 cycles of 98°C for 30 s, 58°C for 30 s, and 72°C for 45 s and a final elongation at 72°C for 2 min. The resulting amplicon replicates were pooled and run on a 1.2% (w/v) agarose gel and positive amplicons were excised from the gel and further purified using the QIAquick Gel Extraction Kit (Qiagen).

Purified amplicon libraries were quantified with Qubit 3.0 Fluorometer and Qubit^®^ dsDNA HS Assay Kit (Thermo Fisher Scientific – Invitrogen, Waltham, MA, United States). All libraries were pooled in an equimolar ratio to 4 nM. Fragment size and library concentration were further confirmed on the Bioanalyzer 2200 TapeStation system (Agilent Technologies, Santa Clara, CA, United States). The normalized library pool was further diluted to 10 pM, spiked with 20% (10 pM) PhiX control and sequenced on an Illumina Miseq platform (Illumina, San Diego, CA, United States) using the MiSeq^®^ reagent kit V3 to generate 2 × 300 base pair reads.

### Sequence Data, Quality Control and Processing

Sequence data were processed and analyzed using USEARCH v9.2.64 (Edgar, 2013) and QIIME v1.9.1 (Caporaso et al., 2010) pipelines. Quality of raw sequencing reads were assessed using FastQC software (Andrews, 2010). The sequence length of the V4 hypervariable region of 16S rRNA gene is approximately 250 base pairs for most microbial species (Kozich et al., 2013), and it was individually covered by both forward and reverse reads. Only forward reads were retained for downstream analysis since their quality was generally better than the corresponding reverse reads. They were trimmed to 260 base pairs; to remove the primer sequence and any subsequent sequences at the 3′ end. Sequences with less than 260 base pairs were removed. A maximum expected error filter strategy with a threshold of 1 was adopted to get rid of the low quality reads (Edgar and Flyvbjerg, 2015). Chimeric sequences were removed using the UCHIME algorithm (Edgar et al., 2011). Then, quality filtered sequences were clustered into operational taxonomic units (OTUs) at 97% sequence similarity threshold. The representative sequences of OTUs were assigned to different taxa using the SINTAX algorithm (Edgar, 2016) employing the 16S rRNA RDP database (v11.5) and choosing a confidence cutoff of 0.5. Subsequently, OTUs with a confidence score <1 at domain level and the 12 OTUs belonging to the phylum Cyanobacteria were removed since they only accounted for 0.0092% of the total reads. All samples were rarefied to 20,000 sequences and rarefaction curves were obtained by plotting the species richness against the number of sequences per sample. The raw 16S sequence data from this study has been deposited at the European Nucleotide Archive database (EBI) under the accession number ERS2484299 to ERS2484358.

### Data Visualization and Statistical Analysis

We executed R codes in RStudio v1.1.442 (R Development Core Team, 2018) and employed the undermentioned packages to analyze the data. Data were imported into R using the package ‘xlsx.’ The package ‘phyloseq’ v1.22.3 (McMurdie and Holmes, 2013) was used to make phyloseq objects. The data visualization was done mainly using ‘ggplot2’ v2.2.1 (Wickham, 2010). Packages ‘iNEXT’ v2.0.12 (Hsieh et al., 2016) and microbiome v1.0.2 (Lahti et al., 2017) were used to create the rarefaction curves and boxplots of diversity indices.

Pearson’s chi-squared followed by Benjamin–Hochberg *post hoc* tests were performed to determine if the proportions of Proteobacteria/Bacteroidetes in the treatment groups of CR and GF fish differed significantly. Significance was set at *P* < 0.05. To determine the alpha diversity of bacterial communities, species richness, Shannon diversity, and Simpson diversity [effective number of species, Jost (2006)], phylogenetic diversity (PD whole tree, Faith and Baker, 2006), core abundance and rare low abundance [relative proportion of core species and least abundant species (Lahti et al., 2017)] were analyzed. The Kruskal–Wallis test and Dunn’s test with the Benjamini–Hochberg FDR correction were used to identify significant differences in the alpha diversity indices of the groups. To assess the beta diversity, principal coordinate analysis was performed on the matrices generated using weighted UniFrac distances (Lozupone and Knight, 2005). The sample sizes of the experimental groups were different, and we checked for the heterogeneity of dispersions using the function betadisper from vegan v2.4-6 by Oksanen et al. (2018). Analysis of similarity (ANOSIM, employing 999 permutations) and Adonis in the R package ‘vegan’ were employed to understand the differences in clustering and the locations of their centroids. We have examined the differentially abundant bacterial communities of the groups by analyzing the data using linear discriminant analysis effect size (LEfSe) (Segata et al., 2011). An alpha value of 0.05 for the factorial Kruskal–Wallis test and a threshold of 3.5 for the logarithmic LDA score were selected to understand the discriminative features.

## Results

### Sterility of GF and CR Zebrafish Larvae

Microbial status or sterility of larvae was confirmed before yeast exposure. There was no bacterial growth on TSA agar plate inoculated with samples from GF larvae and media. Furthermore, we did not observe any bands after PCR amplification of the 16S rRNA from larvae samples until 7 dpf and water/media from GF zebrafish. On the other hand, CR larvae and rearing media samples produced positive amplification of 16S rRNA; for samples collected at all the sampling points (2, 3, 7, and 14 dpf).

### Sequencing Quality

We analyzed the 16S rRNA V4 amplicon sequences of the bacterial communities of 62 samples. Of these, 25 were intestine samples of CR larvae, 20 were those of GF larvae and remaining 17 were of water from the respective treatment groups and zebrafish facility. We obtained a total of 5,150,530 high-quality reads after adapter and quality trimming of 5,650,637 raw sequences (**Supplementary Table S1**). They were clustered into 2095 OTUs at 97% identity threshold; the OTUs represented 399 different taxa, of which 308 were assigned to specific genera. The majority of the rarefaction curves reached the saturation point at 20,000, allowing us to capture most of the underlying diversity (**Supplementary Figure S1**). Hence, 20,000 sequences per sample were considered to be appropriate for further analysis.

### Relative Abundance and Core Microbiota in Zebrafish Larvae

**FIGURE 2:**
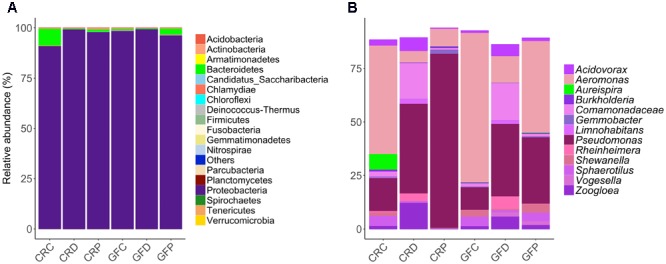
Relative abundance of the intestinal bacterial communities of zebrafish larvae, at different taxonomic levels. Phyla **(A)** and the top abundant genera **(B)** in CR and GF zebrafish. Each bar segment representing the average relative abundance of a particular bacterial taxon within a group is color coded: Proteobacteria – shades of purple and pink, and Bacteroidetes – green. CRC, conventionally raised control; CRD, conventionally raised *Debaryomyces-*exposed; CRP, conventionally raised *Pseudozyma*-exposed; GFC, germ-free control; GFD, germ-free *Debaryomyces*-exposed; GFP, germ-free *Pseudozyma*-exposed.

Analysis of the intestine samples revealed that Proteobacteria (97.8%) was the dominant phylum, followed by Bacteroidetes (1.5%). Actinobacteria, Firmicutes, Fusobacteria and remaining rare phyla accounted for the residual 0.7% of the reads (**Figure 2A**).

The abundance profile at genus level (**Figure 2B**) indicated that the most common genera were present in all larvae groups. Differences between bacterial communities in control and yeast-exposed groups were due to different proportions of these core bacteria as well as some rare genera, which are not shown in **Figure 2B**. The dominant genera, namely *Aeromonas, Acidovorax, Pseudomonas, Rheinheimera, Shewanella, Sphaerotilus, Gemmobacter, Zoogloea* and unidentified genera of the family Comamonadaceae were found among the core microbiota (shared by 97% of the samples) in the zebrafish larvae (**Supplementary Figure S2**). In contrast, some low abundant and rare taxa mainly *Staphylococcus, Photobacterium, Comamonas, Plesiomonas, Vibrio*, and *Cetobacterium* were unique to some samples, but we did not find any considerable correlation of these genera with yeast exposure.

### Yeast-Exposed Larvae Display a Distinct Bacterial Relative Abundance Compared to the Control Larvae

The relative abundance of phylum-level taxa indicates that Proteobacteria is the predominant phylum in all the treatment groups of CR larvae. However, the proportion of Bacteroidetes was higher in CRC (8.8%, *P* = 0) in comparison to *Debaryomyces*-exposed (0.3%) and *Pseudozyma*-exposed (1.1%) CR larvae (**Figure 2A**). These differences were more evident at lower taxonomic levels (**Supplementary Figures S3A–C**). Additionally, the relative abundance of the 20 most abundant taxa revealed notable differences between conventionally raised control (CRC) and yeast-exposed groups (CRD/CRP; **Figure 2B**). At the genus level, *Aeromonas* and *Pseudomonas* were the two predominant taxa. The abundance of *Aeromonas* in yeast-exposed groups decreased, while that of *Pseudomonas* increased compared to the control group (**Figure 2B**). A similar pattern was also observed in the GFC compared to the corresponding yeast-exposed groups (GFD, GFP; **Figures 2A,B**).

**FIGURE 3:**
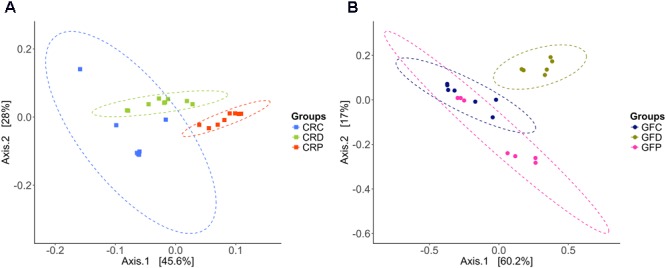
Principal coordinate analysis plots based on weighted UniFrac distance metric show the distinct intestinal bacterial communities in CR **(A)**, and GF **(B)** of zebrafish larvae. Ellipses include 95% of samples from normally distributed data. CRC, conventionally raised control; CRD, conventionally raised *Debaryomyces-*exposed; CRP, conventionally raised *Pseudozyma*-exposed; GFC, germ-free control; GFD, germ-free *Debaryomyces*-exposed; GFP, germ-free *Pseudozyma*-exposed.

The microbial community compositions of CRC and yeast-exposed larvae were significantly different, according to the weighted UniFrac distance-based analysis, which took the bacterial abundance into account rather than presence and absence of taxa. PCoA analysis revealed a clear clustering pattern; samples were largely partitioned based on the yeast exposure (**Figure 3A**, ANOSIM; *R* = 0.80, *P* < 0.001 and Adonis; *R*^2^ = 0.55, *P* < 0.001). Even though axis one captures more variation than axis two, the samples were better separated by axis two. Significant differences between the groups (CRC vs. CRD – *P* < 0.001, CRC vs. CRP – *P* < 0.001 and CRD vs. CRP – *P* < 0.001) were revealed when the groups were considered separately in pairwise comparisons. Samples of the CRC group clustered near the CRD samples, while those of the CRP clustered separately.

Regarding the GF samples, the cluster of the GFD group was distinct compared to GFC and GFP (ANOSIM; *R* = 0.57, *P* < 0.001 and Adonis; *R*^2^ = 0.50, *P* < 0.001, **Figure 3B**). The GFP group clustered closer to GFC, unlike the CRP group of the CR larvae. Significant difference between groups (GFC vs. GFD – *P* < 0.01, GFC vs. GFP – *P* < 0.05 and GFD vs. GFP – *P* < 0.01) were also observed during pairwise comparisons.

### Bacterial Diversity Was Significantly Affected by Exposure to Yeast

**FIGURE 4:**
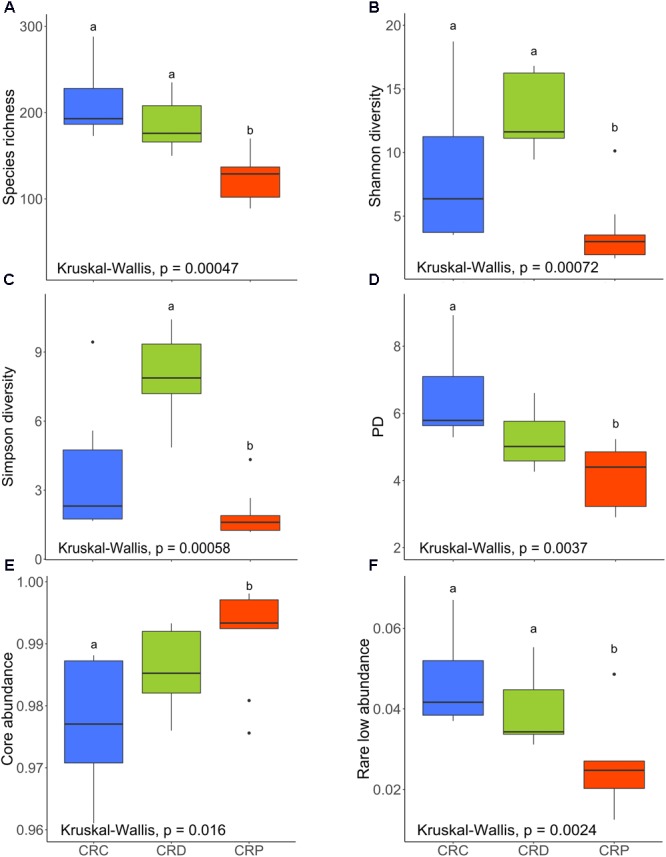
Alpha diversity indices of the intestinal bacterial communities of CR zebrafish larvae. Species richness **(A)**, Shannon diversity **(B)**, Simpson diversity **(C)**, phylogenetic diversity **(D)**, core abundance **(E)**, and rare low abundance **(F)**. Different letters above the bars indicate significant differences as determined by Dunn’s tests. CRC, conventionally raised control; CRD, conventionally raised *Debaryomyces-*exposed; CRP, conventionally raised *Pseudozyma*-exposed.

We did not observe any significant differences in species richness and Shannon diversity indices (**Figures 4A,B**) between CRC and CRD groups (*P* > 0.05) but when we compared *Pseudozyma*-exposed group (CRP) with CRD significant differences were obtained (*P* < 0.01) or CRC (*P* < 0.01). The species richness and evenness of the community was relatively higher (*P* > 0.05) in the CRD compared to the CRP group. Simpson diversity (dominance) of the CRP group was significantly lower when compared to the CRD (*P* < 0.001), whereas no significant difference was observed for the CRD vs. CRC comparison (*P* > 0.05; **Figure 4C**). Microbial phylogenetic diversity decreased as a result of exposure to *Pseudozyma* (CRP); the value was higher in the CRC (*P* < 0.01), but that of the CRD group was not significantly different (*P* > 0.05; **Figure 4D**). The core abundance value of the CRP group was significantly different from the CRC group (*P* < 0.05) but similar to CRD (*P* > 0.05; **Figure 4E**). The rare low abundance of the CRP group was significantly lower than that of CRC (*P* < 0.01) and CRD (*P* < 0.05; **Figure 4F**).

**FIGURE 5:**
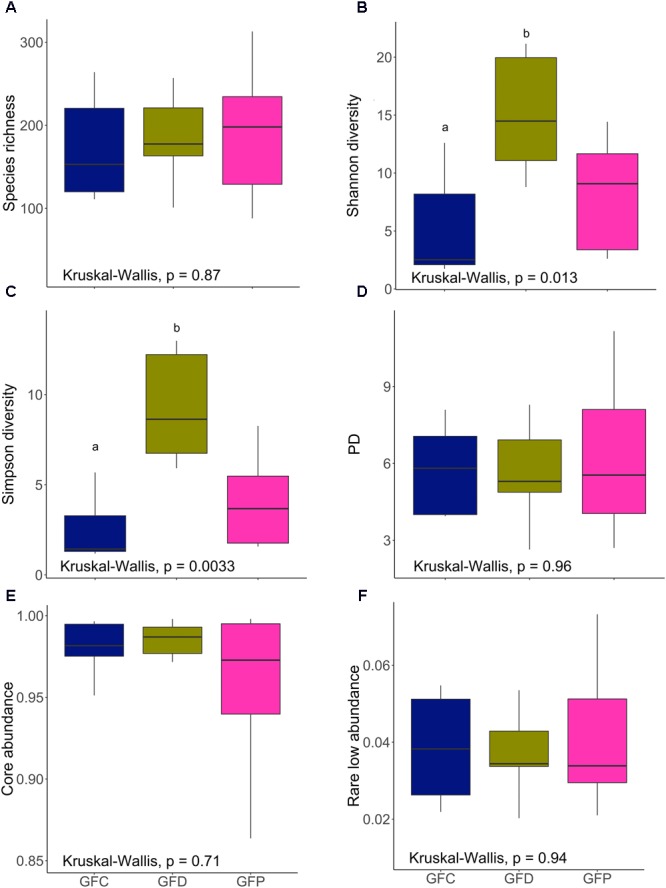
Alpha diversity indices of the intestinal bacterial communities of GF zebrafish larvae. Species richness **(A)**, Shannon diversity **(B)**, Simpson diversity **(C)**, phylogenetic diversity **(D)**, core abundance **(E)**, and rare low abundance **(F)**. Different letters above the bars indicate significant differences as determined by Dunn’s tests. GFC, Germ-free control; GFD, germ-free *Debaryomyces*-exposed; GFP, germ-free *Pseudozyma*-exposed.

In contrast to the results of the CR conditions, *Debaryomyces*-exposed germ-free group (GFD) had significantly higher Shannon and Simpson diversities when compared to the control group (GFC, *P* < 0.01; **Figures 5B,C**). However, Shannon and Simpson diversities of the GFD and GFP groups were not significantly different (*P* > 0.05; **Figures 5B,C**). Exposure to yeasts had no significant effect on microbial alpha diversity measures (**Figures 5A,D–F**), the exception being the abovementioned results of Shannon and Simpson diversity.

### Germ-Free and Conventionally Raised Zebrafish Larvae Not Exposed to Yeast Have Similar Intestinal Microbiota

The compositions of the microbiota in the control larvae reared in GF and CR conditions were almost identical (**Figures 2A,B**). PCoA also did not reveal any significant differences between GF and CR larvae (**Supplementary Figure S4A**, ANOSIM; *R* = 0.01, *P* > 0.05 and Adonis; *R*^2^ = 0.11). Similarly, we have not found any significant differences in the alpha diversity measures of the GF and CR larvae (**Supplementary Figures S4B–F**). Altogether, microbial composition and diversity of GF and CR larvae were similar.

### Differentially Abundant Taxa Differ Between the Treatments

**FIGURE 6:**
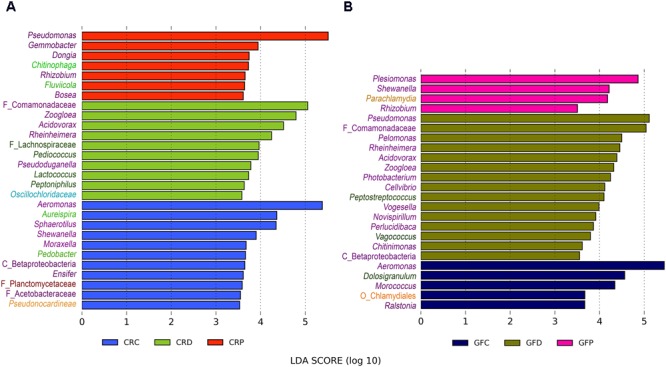
Differentially abundant bacterial taxa in CR **(A)** and GF **(B)** zebrafish larvae intestine. LEfSe was employed to find the differential abundance using a cut-off of 3.5 and a significance threshold of *P* < 0.05. *Y*-axis labels are color coded for different bacterial taxa: Proteobacteria – purple, Bacteroidetes – light green, Firmicutes – dark green, Chlamydiae – coral, Chloroflexi– cyan, Planctomycetes – dark red, and Actinobacteria – light orange. CRC, conventionally raised control; CRD, conventionally raised *Debaryomyces-*exposed; CRP, conventionally raised *Pseudozyma*-exposed; GFC, germ-free control; GFD, germ-free *Debaryomyces*-exposed; GFP, germ-free *Pseudozyma*-exposed.

Linear discriminant analysis effect size analysis revealed that the genus *Aeromonas* was the overrepresented taxon in control zebrafish, regardless of the rearing conditions (**Figures 6A,B**). On the other hand, *Pseudomonas* was observed as the significantly abundant type in the yeast-exposed group, i.e., in the *Pseudozyma-*exposed larvae of the CR group (**Figure 6A**) and *Debaryomyces*-exposed larvae in the GF group (**Figure 6B**), respectively. We also observed that relatively few taxa were differentially abundant in *Pseudozyma*-exposed larvae (7 and 4 taxa in CR and GF, respectively) in comparison to the control (11 and 5 in CR and GF, respectively) and *Debaryomyces*-exposed larvae (10 and 15 in CR and GF, respectively). *Zoogloe, Acidovorax, Rheinheimera* were more abundant in the CRD and GFD groups. *Rhizobium* was overrepresented only in *Pseudozyma*-exposed groups of the CR and GF fish larvae. *Aureispira, Pedobacter, Chitinophaga, Fluviicola* from the second most dominant phylum Bacteroidetes were largely abundant in the CR groups rather than in the GF groups. Bacteria belonging to the phylum Firmicutes, particularly the low abundant *Pediococcus* and *Lactococcus*, were predominant in *Debaryomyces*-exposed CR group. In the GF group, *Peptostreptococcus* and *Vagococcus* were the abundant Firmicutes.

### Bacterial Communities of Water and Intestine of Zebrafish Larvae Differed

**FIGURE 7:**
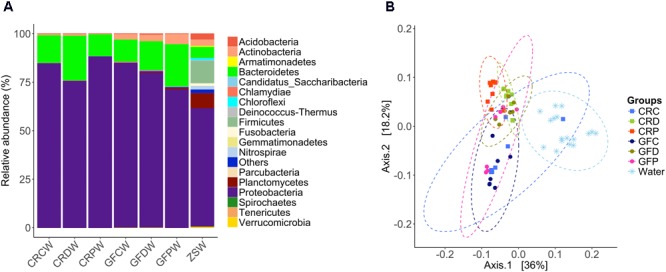
Relative abundance of bacterial phyla in the water samples **(A)** and principal coordinate analysis plot **(B)**. The PCoA plot based on weighted UniFrac distance metric shows the distinction between the bacterial communities in water and zebrafish intestine. Ellipses include 95% of samples from normally distributed data. CRCW, CRDW, and CRPW represent the water samples collected from their respective flasks/tanks on day 2, 3, 7, and 14. GFCW, GFDW, and GFPW represent the water samples collected only on day 14. ZSW represents the water samples collected from the zebrafish facility on days 7 and 14.

Proteobacteria, Bacteroidetes and Actinobacteria were the dominant groups in water samples from the two rearing conditions (CR and GF). These abundant phyla were also the dominant type in the samples from the water supply source of the zebrafish facility. They also contained other dominant phyla, namely Firmicutes, Fusobacteria, Nitrospirae, Planctomycetes, and Verrucomicrobia (**Figure 7A**). At a class level, we observed clear differences in microbial communities between water and intestinal samples (**Supplementary Figure S5**). Similarly, PCoA plot based on weighted UniFrac distance metrics indicated that these groups harbored distinct microbial populations (**Figure 7B**), despite the dominance of the phylum Proteobacteria in both the water and intestine samples.

## Discussion

Early life-associated factors such as diet, environmental conditions, microbial interactions, and exposure to antibiotics or probiotics decide the assemblage and structure of the naïve microbial communities of the hosts (Scholtens et al., 2012; de Muinck et al., 2013). These microbes facilitate the host’s acclimatization to a new environment and subsequent succession of the associated intestinal microbial community. Several studies in most vertebrates, including fish have demonstrated that the selective manipulation through prebiotics and/or probiotics or functional diets modulates the gut microbial community by favoring the growth of beneficial microbes (Montalban-Arques et al., 2015; Ringø et al., 2016; Zorriehzahra et al., 2016). Probiotic administration is a promising approach to maintain the microbial balance, to prevent diseases, and to improve the health status of the host (Kiron, 2015). However, our current knowledge on the influence of yeasts on fish gut microbial composition is based on studies in a few species. We have determined the effect of fish-derived yeasts on the composition of bacteria in the intestine of zebrafish by sequencing the V4 region of bacterial 16S rRNA gene on an Illumina MiSeq platform. Our data demonstrated that early exposure to yeasts shapes the bacterial community and diversity in GF and CR zebrafish larvae and provide evidence that fish derived-yeasts can be used as biological tools to induce alterations in the gut microbial communities.

### Proteobacteria Is the Dominant Phylum in Zebrafish Intestine

Bacterial communities in the intestine of zebrafish larvae comprised Proteobacteria (97.8%), Bacteroidetes (1.5%) and other rare phyla (**Figure 2A**); this profile is similar to other fish gut microbiota, including adult zebrafish (Roeselers et al., 2011; Llewellyn et al., 2014; Stephens et al., 2016). Our result is also consistent with previous reports that zebrafish larval gut harbors a highly constrained set of bacterial phyla (Davis et al., 2016; Phelps et al., 2017; Dahan et al., 2018). At the genus level, *Aeromonas, Acidovorax, Pseudomonas, Rheinheimera, Shewanella, Sphaerotilus, Gemmobacter*, and *Zoogloea*, and unidentified genera of the family Comamonadaceae were the dominant bacteria (**Figure 2B**). Among these, *Aeromonas, Pseudomonas, Shewanella* and few other rare genera (*Staphylococcus, Photobacterium, Comamonas, Plesiomonas, Vibrio*, and *Cetobacterium*) were commonly found and reported as the core gut microbiota of zebrafish (Roeselers et al., 2011; Cantas et al., 2012). The existence of these core communities in the intestine could reflect the significance and functional association of these bacteria with zebrafish. Although we did not find the abovementioned rare genera among the core taxa, this hidden backbone of microbial communities is known to enhance the functionality of abundant microbes (Jousset et al., 2017).

### Yeast Exposure Alters the Relative Abundance of *Aeromonas* and *Pseudomonas*

Zebrafish larvae that were exposed to yeasts had significantly different microbial composition than their counterparts in the control groups (**Figures 3A,B**). Bacteroidetes decreased in the yeast-exposed groups compared to the control (**Figure 2A**). However, the effect of yeasts on the members of Proteobacteria seems to be selective; manifested by a trade-off between the relative abundance of two core genera, *Aeromonas* and *Pseudomonas* (**Figure 2B**). These bacterial genera are natural inhabitants of the aquatic environments and could persist in the fish gut as well (Cantas et al., 2012). Most bacteria belonging to these genera are opportunistic pathogens (Austin and Austin, 2007) but a few species are acknowledged as probiotic candidates in aquaculture (Irianto and Austin, 2002; Pieters et al., 2008; Van Hai and Fotedar, 2009). The intestinal microbiota of the control group was enriched with *Aeromonas*, whereas yeast-exposed larvae were enriched with *Pseudomonas*. Fish-derived yeasts or their cellular components seem to have antagonistic activity against the members of *Aeromonas* but favor the growth of *Pseudomonas* species. Yeasts and bacteria are common colonizers of gastrointestinal tract of animals, and their interaction, either symbiotic or antagonistic (Arvanitis and Mylonakis, 2015), has been shown to play a major role in the host biological activities (Iliev and Underhill, 2013). Furthermore, yeasts and their cell wall components including β-glucans, mannans and chitin may act as prebiotics or probiotics not only to facilitate the growth of beneficial bacteria but also to suppress the growth of other microbes via the production of various metabolites (Hatoum et al., 2012). Cider yeast-supplemented diet modulated the gut microbiota of pigs and reduced the counts of *Lactobacilli* (Upadrasta et al., 2013). Similarly, Gonçalves and Gallardo-Escárate (2017) showed that rainbow trout fed *S. cerevisiae*-supplemented diets had decreased abundance of Gammaproteobacteria of the phylum Proteobacteria and increased abundance of the phyla Firmicutes and Fusobacteria. A higher abundance of *Pseudomonas* in the yeast-exposed group also suggests that they outcompete *Aeromonas*. The antifungal properties of *Pseudomonas* against *Candida* spp. (fungi) have been reported; these bacteria can produce phenazines (Nishanth Kumar et al., 2014) and quorum-sensing molecules (Hogan et al., 2004). Furthermore, *Pseudomonas* has developed a mechanism to restrict *Candida* to its yeast form, so as to take advantage of the yeast fermentation products for its own growth and proliferation. Their complex interaction also involves the attachment of *Pseudomonas* to existing *Candida* hyphae to aid its dissemination (Chen et al., 2014). Conversely, *Candida* is able to down-regulate virulence factors of *P. aeruginosa* by modulating its quinolone signal (Cugini et al., 2007), which is part of the quorum-sensing system and controls multiple virulence factors (Calfee et al., 2001).

### Yeast Exposure Impacts the Intestinal Bacterial Diversity

We have examined the effect of yeast exposure on the bacterial diversity in the intestine of zebrafish larvae. Regarding the CR larvae, *Pseudozyma* had a significant impact on both species richness and diversity indices (**Figures 4A,B**). However, such an effect was not observed for *Debaryomyces*. The lower diversity (richness, common and dominant species) of bacteria in the CRP group compared to CRC and CRD groups may be related to the predominance of *Pseudomonas* in CRP larvae (**Figure 2B**). The intestinal bacteria in the CRP group were phylogenetically more homogenous or closely related than those of the CRC and CRD groups (**Figure 4D**). We also observed a significant difference in the abundance of core community in CRP compared to CRC but not between CRP and CRD (**Figure 4E**). Again, this can be due to higher relative proportion of *Pseudomonas*, which is part of the zebrafish core community and could explain the significantly lower proportion of the low abundant species (**Figure 4F**). As for the germ-free larvae, the GFD group had significantly higher Shannon and Simpson diversity compared to the GFC group, suggesting that exposure to yeast can facilitate the establishment of diverse bacteria in the intestine of germ-free larvae. On the other hand, we did not observe any significant differences among other alpha diversity indices of the GF larvae. In CR zebrafish, the effect of yeast-exposure on the different diversity indices of intestinal bacteria contrasted with previous observations in rainbow trout (Waché et al., 2006; Gonçalves and Gallardo-Escárate, 2017). These authors reported that feeding rainbow trout fry and juveniles with diet containing *S. cerevisiae* increases their gut microbial diversity. Similarly, feeding dietary mannan-oligosaccharides to juvenile rainbow trout and gilthead seabream (*Sparus aurata*) increased the diversity of gut microbiota (Dimitroglou et al., 2010; Gonçalves and Gallardo-Escárate, 2017). Our results are in agreement with a previous report on the reduction of microbial diversity of zebrafish larvae after immersion exposure to probiotic *Lactobacillus rhamnosus* GG (Falcinelli et al., 2016). Interestingly, we have observed increased microbial diversity only in germ-free larvae exposed to *Debaryomyces* (GFD). Tapia-Paniagua et al. (2011) observed lower Shannon and Simpson diversity of the gut microbiota of *S. aurata* after the fish consumed diets incorporated with *D. hansenii* L2 for 4 weeks. The result of immersion versus feeding exposure to probiotics on gut microbiota depends on several factors, including the probiotic strain in question, dosage, viability and ability to cope with alimentary tract conditions in the host (Hai, 2015). In some cases, a similar colonization success is achieved through live feed and rearing water. However, three probiotic strains of *Ruegeria, Pseudoalteromonas*, and *Vibrio* were unable to colonize the gut of Atlantic cod (*Gadus morhua*) larvae either by immersion or feeding exposure (Skjermo et al., 2015). Nevertheless, the influence of yeast exposure methods on gut microbiota in fish warrants further investigation.

Microbial composition, diversity and abundance are positively affected by favorable conditions related to space, nutrients and competition (Hibbing et al., 2010). *Debaryomyces*-exposed CR and GF zebrafish larvae had relatively higher alpha diversity indices and we speculate that it might provide favorable intestinal conditions due to its inherent probiotic properties. For instance, juvenile leopard grouper (*Mycteroperca rosacea*) and gilthead seabream (*S. aurata*) offered live *D. hansenii* CBS8339 and strain L2, respectively, had enhanced immune responses and were resistant to pathogenic *A. hydrophila* (Reyes-Becerril et al., 2011, 2012). In addition, cell wall components of yeast can influence the host immune responses to regulate the diversity of gut microbiota (Charlet et al., 2018). The prebiotic/probiotic efficacy of a yeast is dependent on many factors including the type of species. In our study, larvae exposed to zebrafish-derived *Pseudozyma* had more abundance of *Pseudomonas* and lower microbial diversity compared to *Debaryomyces*, which was isolated from Atlantic salmon. Unlike *Pseudozyma, Debaryomyces* might have positive effect on host by increasing the microbial diversity. Thus, the species of yeast impacts the diversity of bacterial species which in turn influence the bacterial assemblage. Several studies have shown that the presence of diverse microbiota indicate a healthy ecosystem, which increases microbial functional stability and adaptive capacity (De Roy et al., 2013). However, its beneficial effects depend on the presence or enrichment of certain genera/species in the community rather than diversity *per se*. For example, it has been demonstrated that functional characteristics of a small number of bacterial species (low richness) have a positive impact on feed efficiency and energy requirements in ruminants (Shabat et al., 2016).

### Exposure to *Debaryomyces* Favors Beneficial Bacteria

Linear discriminant analysis has indicated that taxa belonging to Proteobacteria were differentially abundant in all the groups (**Figures 6A,B**), irrespective of treatment. However, taxa from the Bacteroidetes were only enriched in CR zebrafish larvae (**Figure 6A**). *Debaryomyces*-exposed groups (GF and CR) were characterized by several significantly abundant bacterial taxa that included beneficial bacteria. Particularly, species from the low occurring phylum Firmicutes including *Pediococcus* and *Lactococcus* were enriched in CRD. Increasing abundance of LAB after yeast supplementation has also been reported in studies of juvenile European sturgeon (Hoseinifar et al., 2011) and Nile tilapia (Ran et al., 2015). Exposure to *Debaryomyces* may improve and maintain homeostasis in the gut ecosystem by modulating the abundance and diversity of bacteria, including those of LAB.

### Variation in the Intestinal Bacterial Assemblage of Zebrafish Larvae Is Not Determined by Their Rearing Conditions

The CR larvae in control group were colonized with commensal bacteria as soon as they hatched from their protective chorions. On the other hand, GF larvae in the control group keep the GF conditions up to 7 dpf. Surprisingly, the microbial composition and diversity in the intestinal tract of both the GF and CR larvae were similar after they were reared in the system tanks (**Supplementary Figure S4A**). The intestinal bacterial composition of zebrafish larvae was distinct from that of the rearing water (**Figure 7B**). Similar results have been observed in other fish studies (Lokesh et al., 2018; Sevellec et al., 2018; Zhang et al., 2018). The intestinal microbiota of zebrafish larvae at 4 and 8 dpf had more abundance of Gammaproteobacteria, whereas the corresponding water samples had predominance of Betaproteobacteria (Stephens et al., 2016). We also observed more abundance of Gammaproteobacteria in the intestine of zebrafish larvae than their rearing water, which was mostly comprised of Betaproteobacteria (**Supplementary Figure S5**).

Our results suggest that exposure to yeast during the early developmental stage of zebrafish could influence the microbiota, and the differences in the established communities were evident after they were maintained in similar rearing conditions. In other words, variation in microbial composition is driven by specific yeast species, rather than the rearing condition of zebrafish larvae. Thus, our observations are in agreement with those of Yan et al. (2012) who proposed that bacterial community assembly and dynamics are shaped by both deterministic and stochastic factors.

## Conclusion

The present study demonstrates that exposure to fish-derived yeast can alter the composition and diversity of the intestinal bacteria of zebrafish larvae. Noticeably, even a transient early-life exposure to yeast could induce remarkable alterations in the bacterial assemblage. If persistent, this early modulation of microbiota could influence host physiology later in life.

## Author Contributions

PS, VK, JF, VM, and JG-V designed the study. PS and JG-V conducted the experiments. VM, JF, and VK contributed reagents and materials for the experiments. PS and JL performed the laboratory work. JL, PS, VK, and JF analyzed the data. PS, VK, and JF wrote the manuscript. All authors read, revised, and approved the manuscript.

## Conflict of Interest Statement

The authors declare that the research was conducted in the absence of any commercial or financial relationships that could be construed as a potential conflict of interest.
