# Supplementary material for: Exposure to Yeast Shapes the Intestinal Bacterial Community Assembly in Zebrafish Larvae
Source: Front Microbiol. 2018 Aug 14;9:1868. doi: 10.3389/fmicb.2018.01868 (PMC6103253; doi:10.3389/fmicb.2018.01868)
Supplement: Supplementary file 1 [file Data_Sheet_1.pdf]

Exposure to yeast shapes the intestinal bacterial community assembly in zebrafish larvae

Prabhugouda Siriyappagouder[1], Jorge Galindo-Villegas[2], Jep Lokesh[1],

Victoriano Mulero[2], Jorge Fernandes*[1] and Viswanath Kiron*[1]

[1]*Faculty of Biosciences and Aquaculture, Nord University, 8049 Bodø, Norway*

[2]*Department of Cell Biology and Histology, Faculty of Biology, Institute of Biomedical Research of Murcia-Arrixaca. Campus Universitario de Espinardo. University of Murcia, 30100 Murcia, Spain*

Table S1. Statistics of raw reads and trimmed reads of all the samples

Sample Id	Raw sequences	After quality trimming
CRC1	54526	50177
CRC2	56431	54390
CRC3	37938	34478
CRC4	48632	44342
CRC5	51821	50317
CRC6	58478	54510
CRC7	51658	45989
CRD1	128488	113093
CRD2	54472	48934
CRD3	75894	68279
CRD4	127404	111224
CRD5	148658	113301
CRD6	123899	106484
CRD7	96223	79999
CRD8	78816	71752
CRD9	90852	80822
CRP1	53603	51498
CRP2	124062	120184
CRP3	126043	121075
CRP4	67475	62295
CRP5	171474	159506
CRP6	43049	39672
CRP7	101108	96184
CRP8	98585	92435
CRP9	114573	108534
GFC3	35949	34162
GFC4	43949	40974
GFC5	43919	40550
GFC6	42610	40292
GFC7	48266	46006
GFC8	42014	36986
GFC9	120002	113035
GFD10	36666	34379
GFD2	67269	57993
GFD3	137165	123530
GFD4	48052	44114
GFD5	79478	73898

GFD6	88528	79983
GFP10	28470	21995
GFP11	53220	47814
GFP12	145222	138178
GFP13	140247	131555
GFP4	30553	28287
GFP5	32560	29966
GFP6	48805	46578
CRC14W	243336	218264
CRC2W	97713	87535
CRC3W	83013	75163
CRC7W	106138	97403
CRD14W	155046	145734
CRD2W	107065	97035
CRD3W	101635	89833
CRD7W	61324	55498
CRP14W	219045	202215
CRP2W	109509	99762
CRP3W	73035	66023
CRP7W	68900	63447
GFC14W	139092	128806
GFD14W	219683	202688
GFP14W	123267	111824
SW14	108891	99781
SW7	136839	119770
**Total**	**5650637**	**5150530**
