# Supplementary material for: Exposure to Yeast Shapes the Intestinal Bacterial Community Assembly in Zebrafish Larvae
Source: Front Microbiol. 2018 Aug 14;9:1868. doi: 10.3389/fmicb.2018.01868 (PMC6103253; doi:10.3389/fmicb.2018.01868)
Supplement: Supplementary file 2 [file Data_Sheet_2.pdf]

Exposure to yeast shapes the intestinal bacterial community assembly in zebrafish larvae

Prabhugouda Siriyappagouder[1], Jorge Galindo-Villegas[2], Jep Lokesh[1],

Victoriano Mulero[2], Jorge Fernandes*[1] and Viswanath Kiron*[1]

[1]*Faculty of Biosciences and Aquaculture, Nord University, 8049 Bodø, Norway*

[2]*Department of Cell Biology and Histology, Faculty of Biology, Institute of Biomedical Research of Murcia-Arrixaca. Campus Universitario de Espinardo. University of Murcia, 30100 Murcia, Spain*

<u>Supplementary figure legends</u>

FIGURE S1. Rarefaction curves for the species richness of the intestinal bacteria in zebrafish larvae. The curves were generated by plotting the number of species against the number of sequences per sample. The shaded portion around each line represents the 95% confidence interval. Colour codes and the respective samples are: Royal blue – CRC (n = 7), yellow green – CRD (n = 9), orange red – CRP (n = 9), navy blue – GFC (n = 7), yellow – GFD (n = 6), maroon – GFP (n = 7) and sky blue – water samples (n = 17, from all the treatments and zebrafish facility).

FIGURE S2. Relative abundance of the core microbiota in the intestine of zebrafish larvae. Prevalence and detection thresholds of 0.97 and 0.2, respectively were used to determine the core microbiota. Conventionally-raised control– CRC, conventionally-raised *Debaryomyces*-exposed– CRD, conventionally-raised *Pseudozyma*-exposed– CRP, germ-free control– GFC, germ-free *Debaryomyces*-exposed– GFD, and germ-free *Pseudozyma*-exposed– GFP.

FIGURE S3. Relative abundance of the top 20 abundant taxa (OTUs) among the intestinal bacterial communities of zebrafish larvae. Classes (A), orders (B) and families (C). Shades of purple and pink are given for the phylum Proteobacteria, and green for Bacteriodetes. Conventionally-raised control– CRC, conventionally-raised *Debaryomyces*-exposed– CRD, conventionally-raised *Pseudozyma*-exposed– CRP, germ-free control– GFC, germ-free *Debaryomyces*-exposed– GFD, and germ-free *Pseudozyma*-exposed– GFP.

FIGURE S4. Composition and diversity of the intestinal bacterial communities of the conventionally-raised (CR) and germ-free (GF) zebrafish larvae from the control group. Principal coordinate analysis plot based on weighted UniFrac distance metric (A). Ellipses are drawn include 95% of samples from a normally distributed data. Species richness (B), Shannon diversity (C), Simpson diversity (D), phylogenetic diversity (E) and core abundance (F).

FIGURE S5. Relative abundance of bacterial classes in the intestine of zebrafish larvae and water samples. CRCW, CRDW and CRPW represent the water samples collected from their respective flasks/tanks on day 2, 3, 7 and 14. GFCW, GFDW and GFPW represent the water samples collected only on day 14. ZSW represents the water samples collected from the zebrafish facility on day 7 and 14.

Figure S1.

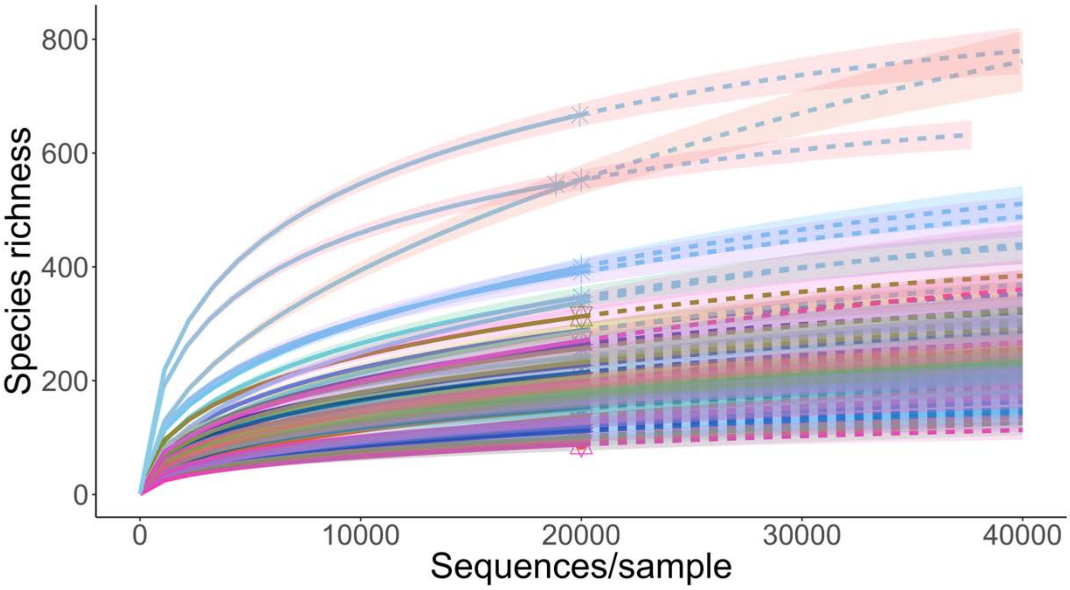
800
600
400
200
0
Species richness
0
10000
20000
30000
40000
Sequences/sample

Figure S2.

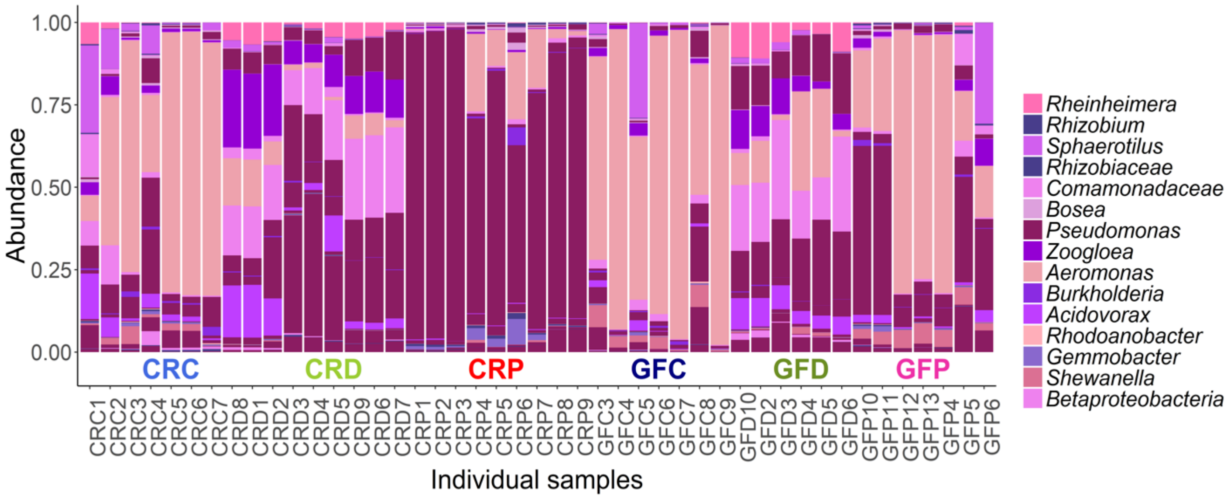

1.00
0.75
0.50
0.25
0.00
Abundance
CRC
CRD
CRP
GFC
GFD
GFP
CRC1
CRC2
CRC3
CRC4
CRC5
CRC6
CRC7
CRD8
CRD1
CRD2
CRD3
CRD4
CRD5
CRD9
CRD6
CRD7
CRP1
CRP2
CRP3
CRP4
CRP5
CRP6
CRP7
CRP8
CRP9
GFC3
GFC4
GFC5
GFC6
GFC7
GFC8
GFC9
GFD10
GFD2
GFD3
GFD4
GFD5
GFD6
GFP10
GFP11
GFP12
GFP13
GFP4
GFP5
GFP6
Individual samples
Rheinheimera
Rhizobium
Sphaerotilus
Rhizobiaceae
Comamonadaceae
Bosea
Pseudomonas
Zoogloea
Aeromonas
Burkholderia
Acidovorax
Rhodoanobacter
Gemmobacter
Shewanella
Betaproteobacteria

Figure S3.

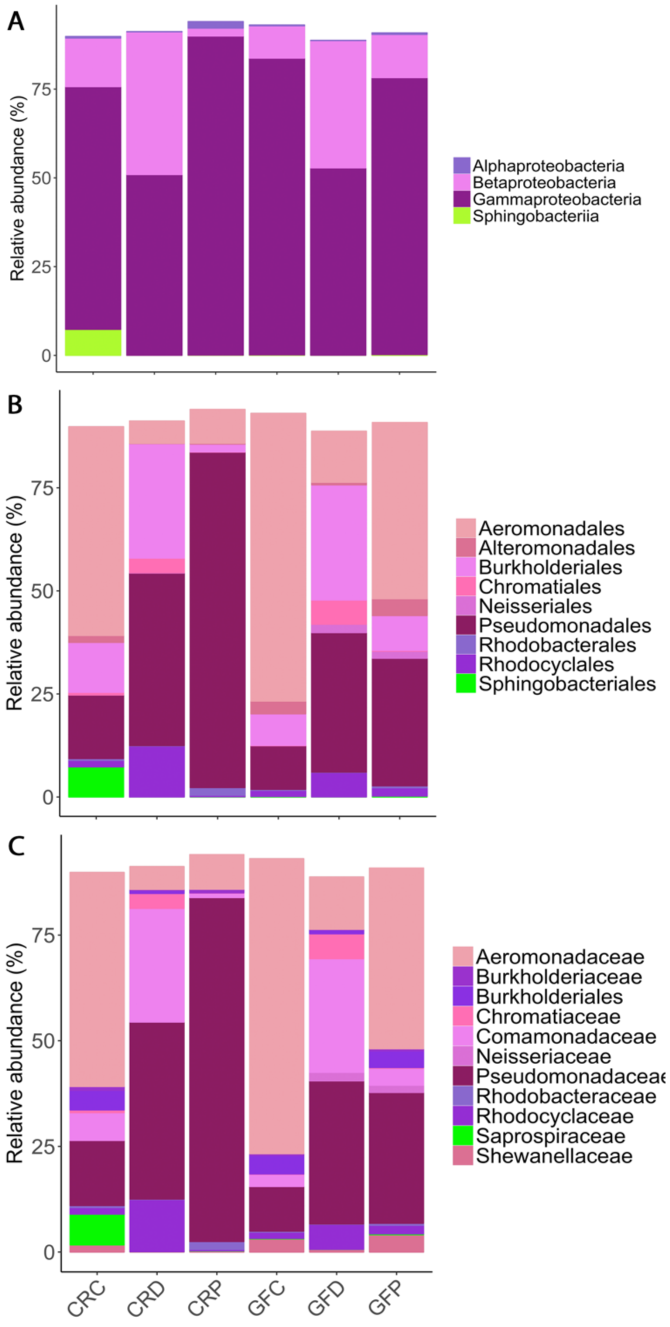
A
B
C
Relative abundance (%)
0
25
50
75
Alphaproteobacteria
Betaproteobacteria
Gammaproteobacteria
Sphingobacteriia
Aeromonadales
Alteromonadales
Burkholderiales
Chromatiales
Neisseriales
Pseudomonadales
Rhodobacterales
Rhodocyclales
Sphingobacteriales
Aeromonadaceae
Burkholderiaceae
Burkholderiales
Chromatiaceae
Comamonadaceae
Neisseriaceae
Pseudomonadaceae
Rhodobacteraceae
Rhodocyclaceae
Saprospiraceae
Shewanellaceae
CRC
CRD
CRP
GFC
GFD
GFP

Figure S4.

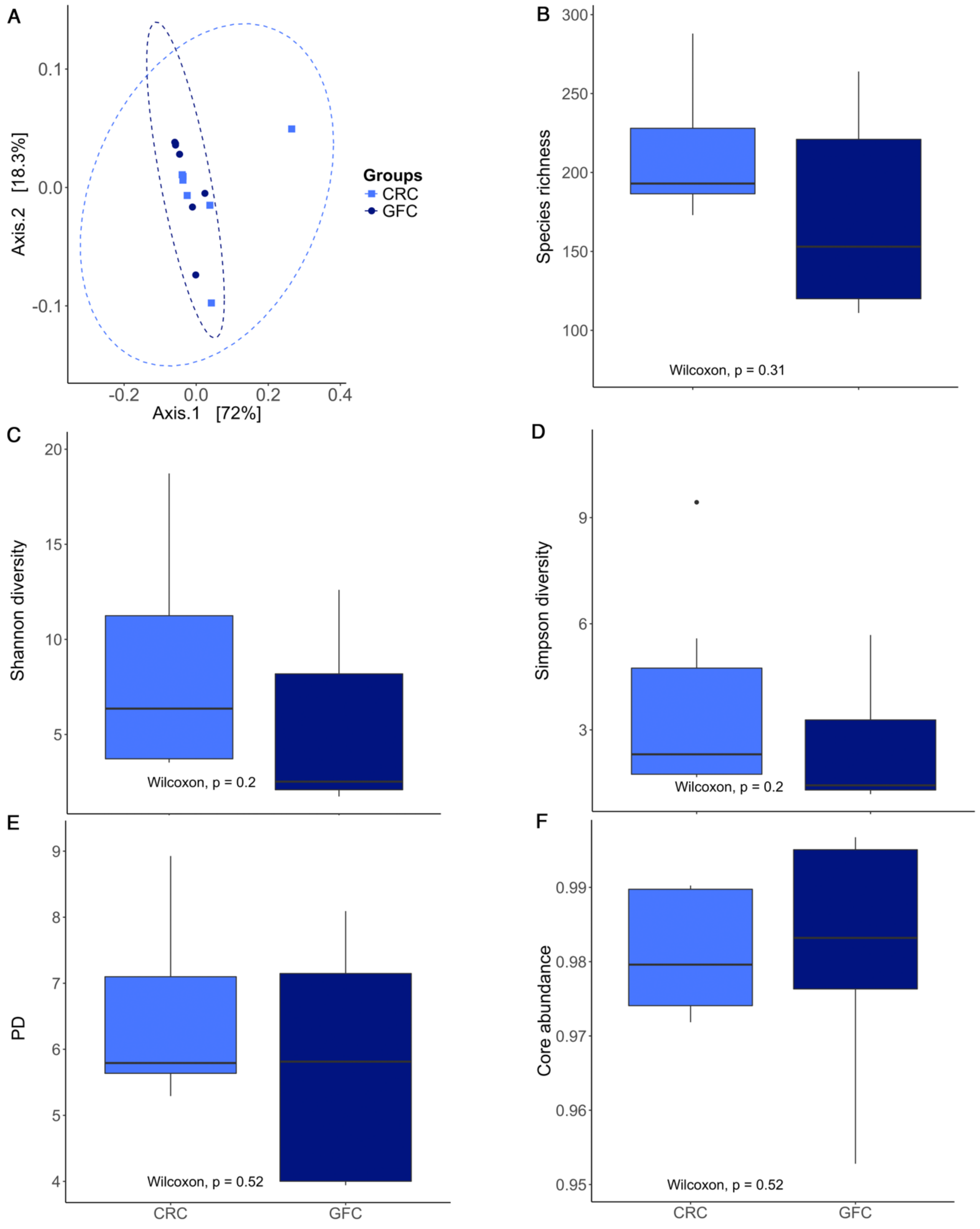
A
Axis.2 [18.3%]
Axis.1 [72%]
0.1
0.0
-0.1
-0.2
0.0
0.2
0.4
Groups
CRC
GFC
B
Species richness
300
250
200
150
100
Wilcoxon, p = 0.31
C
Shannon diversity
20
15
10
5
Wilcoxon, p = 0.2
D
Simpson diversity
9
6
3
Wilcoxon, p = 0.2
E
PD
9
8
7
6
5
4
Wilcoxon, p = 0.52
CRC
GFC
F
Core abundance
0.99
0.98
0.97
0.96
0.95
Wilcoxon, p = 0.52
CRC
GFC

Figure S5.

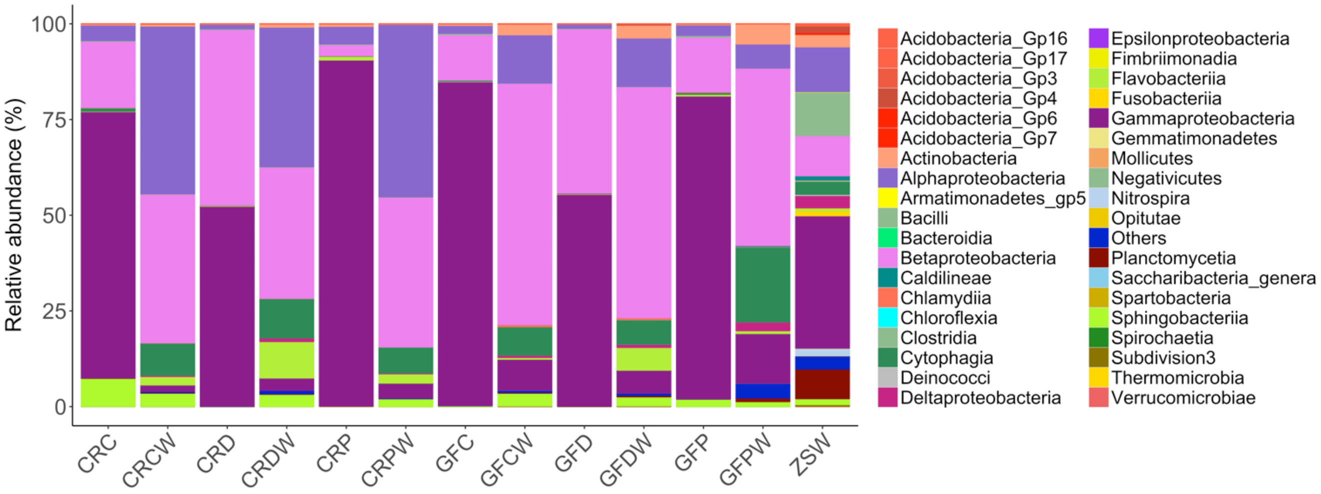
Relative abundance (%)
100
75
50
25
0
CRC
CRCW
CRD
CRDW
CRP
CRPW
GFC
GFCW
GFD
GFDW
GFP
GFPW
ZSW
Acidobacteria_Gp16
Acidobacteria_Gp17
Acidobacteria_Gp3
Acidobacteria_Gp4
Acidobacteria_Gp6
Acidobacteria_Gp7
Actinobacteria
Alphaproteobacteria
Armatimonadetes_gp5
Bacilli
Bacteroidia
Betaproteobacteria
Caldilineae
Chlamydiia
Chloroflexia
Clostridia
Cytophagia
Deinococci
Deltaproteobacteria
Epsilonproteobacteria
Fimbriimonadia
Flavobacteriia
Fusobacteriia
Gammaproteobacteria
Gemmatimonadetes
Mollicutes
Negativicutes
Nitrospira
Opitutae
Others
Planctomycetia
Saccharibacteria_genera
Spartobacteria
Sphingobacteriia
Spirochaetia
Subdivision3
Thermomicrobia
Verrucomicrobiae
